# Can Spinal Bupivacaine Analgesia Treatment Make a Difference on Urinary Bladder Healing According to the Intramuscular Pethidine Analgesia Treatment in Rats?

**DOI:** 10.1100/tsw.2007.284

**Published:** 2007-11-26

**Authors:** Yeswim Senayli, Atilla Senayli, R. Dogan Koseoglu, Ziya Kaya, Fatih Özkan, Ilker Etikan

**Affiliations:** ^1^ Anesthesiology and Reanimation Department , Gaziosmanpaşa University, Medicine Faculty, Tokat, Turkey; ^2^ Pediatric Surgery Department, Gaziosmanpaşa University, Medicine Faculty, Tokat, Turkey; ^3^ Pathology Department, Gaziosmanpaşa University, Medicine Faculty, Tokat, Turkey; ^4^ Biostatistics Department, Gaziosmanpaşa University, Medicine Faculty, Tokat, Turkey

**Keywords:** intrathecal, intramuscular, opiate, meperidin, local anesthetics, bupivacaine, healing, rat

## Abstract

We designed a study to compare the healing levels found with intramuscular pethidine with those found with intrathecal local anesthetic treatments. The urinary bladder is suggested to be the most useful tissue in the evaluation of the effects of the drugs. Nineteen male, Sprague-Dawley rats weighing 200–300 g were used in this study. A sagittal section was made in the urinary bladder after suitable anesthesia and laparotomy. Bladders were closed with 5-0 plain catguts 5 min later. There were nine rats in the control group and pethidine (0.5 g/kg) was administered intramuscularly in the gluteal muscle region to treat pain after the operations. There were 11 rats in the study group and each received a spinal injection of 0.25% bupivacaine after the operation. Rats were followed for 7 days to define pain. Specimens, particularly the incised region of the bladder, were evaluated for inflammation and fibrosis. Grading scales were used for this purpose. Statistical analyses of the data were performed using the Chi-square test. Statistical analyses were nonsignificant for inflammation (*p* ≤ 0.151) and nonsignificant for fibrosis (*p* ≤ 0.105). The treatments may have the same effects on organ healing mechanisms. Statistical difference is not shown in this study, but use of other combinations of pain treatments to evaluate the healing may demonstrate which of these possibilities is true.

## INTRODUCTION

The prevention of pain is important for successful treatment after an operation[1,2]. Thus, if pain occurs, it may impair healing. To prevent this possibility, suitable pain treatment regimens are necessary.

Analgesics and analgesia mechanisms have been studied in clinical trials, and different types of pain management modalities and drugs are found to be suitable for this purpose. One treatment modality is to use opiates intrathecally. Intrathecal opiates act on superficial dorsal horn opiate receptors of the spinal cord[3]. In addition, parenteral administration of opiates activates specific receptors that interfere with transmission of the pain stimulations to the spinal cord and brain[4].

Analgesics, such as local anesthetics, reduce the neuroendocrine stress response, and alleviate somatosensory and visceral pain in the caudal portion of the body[1]. Peripheral nerve blocks, such as epidural blocks, produce sensory and motor blocks; however, they can be hazardous[5]. Urinary retention may be detected with local anesthetic blocks[6]. It was also shown in a previous study that local anesthetics have direct effects on activity of the detrussor muscle[7].

Consequently, both opiates and local anesthetics have direct and indirect effects on bladder activity. We designed a study involving operations on rat bladders and compared the healing levels found with intramuscular pethidine with those found with intrathecal local anesthetic treatments. We evaluated the levels of inflammation and fibrosis in the bladder. We aimed to compare the rates of healing of an operated organ, the urinary bladder, with two different types of analgesia. To our knowledge, our study is the first experimental study to compare the postoperation organ healing levels associated with two different analgesic regimens.

## MATERIAL AND METHODS

The experimental protocol was approved by the Institutional Animal Ethics Committee. Nineteen male, Sprague-Dawley rats weighing 200–300 g were used in this study. The animals were housed and maintained on a 12-h light-dark cycle under ideal conditions (25 ± 0.5°C) with free access to food and water. All animals were neurologically intact before anesthesia and surgery. The study included a control and a study group. Each group underwent the same operation, but the analgesic methods were different.

### Operation

Anesthesia was induced with intravenous ketamine (5 mg/kg) and intraperitoneal xylazine (10 mg/kg) was used for deep anesthesia.

After hair trimming and scrubbing with povidone-iodine, a 2-cm suprapubic transverse incision was performed and the bladder was externalized. A sagittal section was made in the bladder. Bladders were closed with 5-0 plain catguts 5 min later. The abdomen was closed using the same sutures. Cefazolin 20 mg/kg was injected for microbial profilaxy. Rats were observed for 1 week to assess nutrition, movements, pain, and infection. The total manipulation times were 25 ± 4 min for the control group and 32 ± 2 min for the study group.

### Control Group

There were nine rats in the control group and pethidine (0.5 g/kg) was administered intramuscularly in the gluteal muscle region to treat pain after the operations. Rats were followed for 7 days. Pethidine was administered at the same dose for 3 days routinely.

### Study Group

There were 11 rats in the study group and each received a spinal injection of 0.25% bupivacaine after the operation. Spinal catheterization was performed with surgical positioning of the 24G intravenous catheter at the sixth lumbar and first sacral level. The catheter was fixed and buried under the skin suitable for injection, but safe enough to prevent deconnection. Then, 20 µl 0.25% bupivacaine was injected very slowly. Efficiency of the spinal anesthesia and intramuscular analgesia was detected using the tail-flick test and monitoring of hind-leg movement. In all rats, spinal local anesthetics were used for 3 days routinely.

## Evaluation of Bladders

All the rats were sacrificed 7 days after the operations by administration of an overdose of pentobarbital anesthetic, and bladders were excised for analysis by light microscopy with neutral formalin, dehydrated, and embedded in paraffin. Specimens, particularly the incised region of the bladder, were evaluated according to the method described by Hooker et al.[8]. In this method, inflammation and fibrosis were evaluated and grading scales were used.

To assess fibrosis, a 4-point grading scale was used: 0 indicated no fibrosis, 1 indicated minimal and loose fibrosis, 2 indicated moderate fibrosis, and 3 indicated florid and dense fibrosis. A 4-point grading scale for inflammation was also used: 0 indicated no amount of inflammation; 1 indicated giant cells, occasional lymphocytes, and plasma cells; 2 indicated giant cells, plasma cells, eosinophils, and neutrophils; and 3 indicated many inflammatory cells and microabscesses.

## Statistics

Statistical analyses of the data were performed using the Chi-square test. A $p$ value $<0.05$ was considered statistically significant.

## RESULTS

When inflammation was assessed, the control group had no bladders that were grade 0 or 1, five were grade 2, and four were grade 3. In the study group, no bladders were grade 0, one was grade 1, nine were grade 2, and one was grade 3. Statistical analyses were nonsignificant ($p \leq 0.151$) (Table 1).

**TABLE 1**
**Inflammation Grades of the Study and Control Group**

| Inflammation | Grade 0 | Grade 1 | Grade 2 | Grade 3 |
|---|---|---|---|---|
| Control | 0 | 0 | 5 | 4 |
| Study | 0 | 1 | 9 | 1 |
| Statistics (Chi-square) | | $p \leq 0.151$* | | |

* No statistically significant difference.

When fibrosis was assessed, the control group had no bladders that were grade 0, two were grade 1, one was grade 2, and six were grade 3. In the study group, no bladders were grade 0 or 1, five were grade 2, and six were grade 3. Statistical analyses were nonsignificant ($p \leq 0.105$) (Table 2).

**TABLE 2**
**Fibrosis Grades of the Study and Control Group**

| Fibrosis | Grade 0 | Grade 1 | Grade 2 | Grade 3 |
|---|---|---|---|---|
| Control | 0 | 2 | 1 | 6 |
| Study | 0 | 0 | 5 | 6 |
| Statistics (Chi-square) | | $p \leq 0.105$* | | |

* No statistically significant difference.

## DISCUSSION

Visceral pain, as a clinical form of visceral sensation, has been widely investigated[9]. Pain is a stressor and increased production of catecholamines, such as cortisol and other hormones, after anesthesia, surgery, and postoperative pain is known as the neuroendocrine stress response[1]. At the site of injury, processes are initiated, such as platelet aggregation, clot formation, dilatation of blood vessels, and inflammatory cell migration[1]. During wound healing, myofibroblasts are transiently present at the wound site[10]. Stimulation of afferent somatic and autonomic nerves at the wound site is the most important trigger of the neuroendocrine stress response to surgery or trauma[1]. For all of these reasons, management of the pain may improve wound healing. However, as drugs affect organs through several different pathways, pain management may also be hazardous. As no clinical or experimental study has evaluated the effects of pain management regimens on wound healing, in our experimental study, we aimed to compare two different types of pain treatments after urinary bladder operations. One group received spinal local anesthetics to simulate the most preferred treatment after subumbilical operations in adults and sometimes children. The other group received intramuscular pethidine for short-duration analgesic treatment.

Although the mechanism by which opiates cause urinary retention is not completely understood, studies have demonstrated that opiates can inhibit reflex activity of the urinary bladder[11]. Most of these studies were performed in experimental animals and it was shown that opiates suppress micturition in conscious rats after systemic or intrathecal administration. Three opiate receptor subtypes – $\delta$, $\mu$, and $\kappa$ – have been shown to be involved in analgesia, and all three receptors are important for bladder control and micturition events[12].

The intermittent use of intramuscular analgesics is the traditional treatment method of postoperative pain relief for pediatric patients[13]. This method often results in inadequate analgesia for many reasons, including fear of respiratory depression from opiate analgesics and anxieties over opiate addiction. As the potency of meperidine is 10 times less than that of morphine, it is suitable for intramuscular analgesia. Meperidine is a synthetic narcotic analgesic that undergoes hepatic metabolism[14]. Meperidine acts within 15–30 min, with a peak effect occurring after 45–60 min, and acts for up to 4 h[14]. Respiratory and central nervous system depression, seizures, nausea, and vomiting are the usual side effects associated with meperidine. Urinary effects or complications with pethidine have not yet been significantly reported. Although urinary retention with pethidine has not been reported, as it is an opiate, urinary effects would be expected. We thought that characteristics of the drug were similar to intrathecal bupivacaine treatment and pethidine could be compared with spinal analgesia with local anesthetics.

Local anesthetics are widely used to provide regional anesthesia, postoperative analgesia, and treatment of chronic pain conditions. These drugs produce epidural or spinal anesthesia on the mixed nerves in the paravertebral spaces after their passage through the intervertebral foramina, on the dorsal root ganglia (DRG), on the intradural spinal roots, and within the spinal cord[15]. Of these neurons, DRG neurons are key elements in the transmission of sensory stimuli to the central nervous system. It is believed that small myelinated A$\delta$ fibers and unmyelinated C-fibers arise from small DRG neurons and transmit nociceptive sensations[15]. C-fibers are efferent fibers that facilitate reflex and voluntary control of spinal and supraspinal micturition reflexes[12]. In day-case surgeries, the risk of urinary retention is an important reason to withhold regional anesthesia because this can delay hospital discharge. Bupivacaine does not seem to cause transient neurologic symptoms, but its long duration of action is a major disadvantage for its use in day-case surgery[6].

In an *in vitro* study, it was shown that local anesthetics have inhibitory effects on intramural nerves and bladder smooth muscle, so local anesthetics also have direct effects[7]. After injection of the local anesthetics, smaller, unmyelinated sensory fibers like afferent C-fibers on bladder musculature are affected and urodynamics changes are seen[7]. Local anesthetics may be detected in the plasma because of the kinetic processes of the elimination after intrathecal injections[16]. Bupivacaine can be detected for 120 min after administration in the aqueous solution[16]. Thus, intrathecal administration of the local anesthetics can be a risk factor for a complication of urinary bladder dysfunction because of the plasma concentration by the inhibitory effect on nerves and smooth muscle.

Opiates and local anesthetics have direct and indirect effects on healing of organs. Many factors can affect wound healing, but these can be controlled in an experimental study such as this one. The operations and spinal anesthetic manipulations were performed by the same surgeon and anesthetist. Thus, pain management times were not different. All the rats were treated with antimicrobial agents and pain was evaluated.

Statistical significance was not found. Thus, the two modalities are likely to have the same effect on tissues, or alternatively, they did not have negative effects on the urinary bladder. For inflammation and fibrosis, grade 2 results were interesting in the control and study groups. For inflammation, there were five rats in control and nine in study, and for fibrosis, one rat in the control and five rats in the study group. It could be suggested that fibrosis and inflammation in the study group was more than the control group. However, grade 2 findings cannot be defined as a hazardous effect of spinal anesthesia itself, but it can be evaluated in some other more-detailed studies.

This is the first report to compare the effects of two different pain management strategies on healing mechanisms. Thus, it can be said that experimental or clinical experience are insufficient to define differences between the regimens in this study.

In short, we found that one of the spinal bupivacaine or intramuscular meperidine treatments for analgesia could not be selected over the other for a reason such as “good effect on healing” because there was no statistical difference between the two treatments. This may be because the treatments had the same effects on organ healing mechanisms, or it may be that neither treatment affects organ healing mechanisms. Use of other combinations of treatments may demonstrate which of these possibilities is true.

## References

1. Sibanda, S., Hughes, J.M.L., Pawson, P.E., Kelly, G., and Bellenger, C.R. (2006) The effects of preoperative extradural bupivacaine and morphine on the stress response in dogs undergoing femoro-tibial joint surgery. *Vet. Anaesth. Analg.* **33,** 246–257.
2. Yorukoglu, D., Ates, Y., Temiz, H., Yamali, H., and Kecik, Y. (2005) Comparison of low-dose intrathecal and epidural morphine and bupivacaine infiltration for postoperative pain control after surgery for lumbar disc disease. *J. Neurosurg. Anesthesiol.* **17,** 129–133.
3. Pandita, R.K., Pehrson, R., Christoph, T., Friderichs, E., and Andersson, K.E. (2003) Actions of tramadol on micturition in awake, freely moving rats.*Br. J. Pharmacol.* **139,** 741–748.
4. Matsumoto, S., Levendusky, M.C., Longhursta, P.A., Levin, R.M., and Millington, R.W. (2004) Activation of mu opioid receptors in the ventrolateral periaqueductal gray inhibits reflex micturition in anesthetized rats. *Neurosci. Lett.* **363,** 116–119.
5. Edwards, J.L., Pandit, H., and Popat, M. (2006) Perioperative analgesia: a factor in the development of heel pressure ulcers. *Br. J. Nurs.* **15,** 20–26.
6. Breebaart, M.B., Vercauteren, M.P., Hoffmann, V.L., and Adriaensen, H.A. (2003) Urinary bladder scanning after day-case arthroscopy under spinal anaesthesia: comparison between lidocaine, ropivacaine, and levobupivacaine. *Br. J. Anaesth.* **90,** 309–313.

7. Oh, S.J., Kim, S.J., Park, E.C., Chung, H.K., Kim, K.W., and Choi, H. (2001) Effects of local anesthetics on the contractility of rat bladders. *J. Urol.* **165,** 2044–2050.
8. Hooker, G.D., Taylor, B.M., and Driman, D.K. (1999) Prevention of adhesion formation with use of sodium hyaluronate-based bioresorbable membrane in a rat model ventral hernia repair with polypropylene mesh--a randomized, controlled study. *Surgery* **125,** 211–216.
9. Ness, T.J. and Castroman, P. (2001) Evidence for two populations of rat spinal dorsal horn neurons excited by urinary bladder distension. *Brain Res.* **923,** 147–156.
10. Grotendorst, G.R., Rahmanie, H., and Duncan, M.R. (2004) Combinational signaling pathways determine fibroblast proliferation and myofibroblast differentiation. *FASEB J.* **18,** 469–479.
11. Kuipers, P.W., Kamphuis, E.T., van Venrooij, G.E., van Roy, J.P., Ionescu, T.I., Knape, J.T., and Kalkman, C.J. **(**2004) Intrathecal opioids and lower urinary tract function: a urodynamic evaluation. *Anesthesiology* **100,** 1497–1503.
12. Holt, J.D.S., Watson, M.J., Chang, J.P., O'Neill, S.J., Wei, K., Pendergast, W., Gengo, P.J., and Chang, K.J. (2005) DPI-221 [4-((α-*S*)- α-((2*S*,5*R*)-2,5-dimethyl-4-(3-fluorobenzyl)-1-piperazinyl)benzyl)-*N*,*N*-diethylbenzamide]: a novel nonpeptide delta receptor agonist producing increased micturition interval in normal rats. *J. Pharmacol. Exp. Ther.* **315,** 601–608.
13. Yildiz, K., Tezcan, E., Dogru, K., Ozkan, U., and Boyaci, A. (2003) Comparison of patient-controlled analgesia with and without a background infusion after appendicectomy in children. *Paediatr. Anaesth*. **13,** 427–431.
14. Ali, S., Davidson, L.D., and Gremse, D.A. (2004) Comparison of fentanyl versus meperidine for analgesia in pediatric gastrointestinal endoscopy. *Dig. Dis. Sci*. **49,** 888–891.
15. Oda, A., Ohashi, H., Komori, S., Iida, H., and Dohi, S. (2000) Characteristics of ropivacaine block of $Na^+$ channels in rat dorsal root ganglion neurons. *Anesth. Analg*. **91,** 1213–1220.
16. Lazaro, J.J., Franquelo, C., Navarro, X., Castenallo, B., Verdu, E., Cristofol, C., and Arboix, M. (1999) Prolongation of nerve and epidural anesthetic blockade by bupivacaine in a lipid emulsion. *Anesth. Analg*. **89,** 121–127.
